# Biofilm Formation and Genetic Diversity of Microbial Communities in Anaerobic Batch Reactor with Polylactide (PLA) Addition

**DOI:** 10.3390/ijms241210042

**Published:** 2023-06-12

**Authors:** Agnieszka A. Pilarska, Anna Marzec-Grządziel, Emil Paluch, Krzysztof Pilarski, Agnieszka Wolna-Maruwka, Adrianna Kubiak, Tomasz Kałuża, Tomasz Kulupa

**Affiliations:** 1 Department of Hydraulic and Sanitary Engineering, Poznań University of Life Sciences, Piątkowska 94A, 60-649 Poznan, Poland; tomasz.kaluza@up.poznan.pl (T.K.); tomasz.kulupa@gmail.com (T.K.); 2 Department of Agriculture Microbiology, Institute of Soil Science and Plant Cultivation—State Research Institute, Czartoryskich 8, 24-100 Pulawy, Poland; agrzadziel@iung.pulawy.pl; 3 Department of Microbiology, Faculty of Medicine, Wroclaw Medical University, Tytusa Chałubińskiego 4, 50-376 Wroclaw, Poland; emil.paluch@umw.edu.pl; 4 Department of Biosystems Engineering, Poznań University of Life Sciences, Wojska Polskiego 50, 60-627 Poznan, Poland; pilarski@up.poznan.pl; 5 Department of Soil Science and Microbiology, Poznań University of Life Sciences, Szydłowska 50, 60-656 Poznan, Poland; amaruwka@up.poznan.pl (A.W.-M.); adrianna_kubiak@interia.pl (A.K.)

**Keywords:** anaerobic batch reactors, polylactide, genetic diversity, biofilm formation, bacterial morphology

## Abstract

In this paper, an anaerobic digestion (AD) study was conducted on confectionery waste with granular polylactide (PLA) as a cell carrier. Digested sewage sludge (SS) served as the inoculum and buffering agent of systems. This article shows the results of the analyses of the key experimental properties of PLA, i.e., morphological characteristics of the microstructure, chemical composition and thermal stability of the biopolymer. The evaluation of quantitative and qualitative changes in the genetic diversity of bacterial communities, performed using the state-of-the-art next generation sequencing (NGS) technique, revealed that the material significantly enhanced bacterial proliferation; however, it does not change microbiome biodiversity, as also confirmed via statistical analysis. More intense microbial proliferation (compared to the control sample, without PLA and not digested, CW–control, CW–confectionery waste) may be indicative of the dual role of the biopolymer—support and medium. Actinobacteria (34.87%) were the most abundant cluster in the CW–control, while the most dominant cluster in digested samples was firmicutes: in the sample without the addition of the carrier (CW–dig.) it was 68.27%, and in the sample with the addition of the carrier (CW + PLA) it was only 26.45%, comparable to the control sample (CW–control)—19.45%. Interestingly, the number of proteobacteria decreased in the CW–dig. sample (17.47%), but increased in the CW + PLA sample (39.82%) compared to the CW–control sample (32.70%). The analysis of biofilm formation dynamics using the BioFlux microfluidic system shows a significantly faster growth of the biofilm surface area for the CW + PLA sample. This information was complemented by observations of the morphological characteristics of the microorganisms using fluorescence microscopy. The images of the CW + PLA sample showed carrier sections covered with microbial consortia.

## 1. Introduction

The rapidly increasing worldwide consumption of petroleum-based plastics and the depletion of the feedstock have led to a search for materials of natural origin that can contribute to components with properties similar to those of conventional polymers. Ongoing research is oriented towards the search for biopolymers that degrade rapidly in the environment [1,2] while exhibiting beneficial mechanical and physicochemical properties.

Polylactide (PLA) is one of the biopolymers that meet the aforementioned criteria and is currently a very well-known thermoplastic polyester. As a result of its relatively high mechanical strength (flexural strength up to 140 MPa, Young’s modulus 5–10 GPa), excellent optical properties, and good processing ability (with low shrinkage not causing product deformation), PLA is considered the most promising material for many various applications [3,4]. Initially, due to its high production costs, PLA was only used in medical applications [5,6]. However, thanks to the development of technologies for producing PLA and its composites with defined dispersion and thermo-mechanical properties, it is now possible to use PLA in a wide variety of industries, including the food industry, automotive industry, packaging industry, textile industry, agriculture industry, construction industry and others [7,8,9]. Such numerous possibilities of PLA application lead to the generation of its waste.

In times of energy crisis, it makes sense to develop technologies based on renewable energy sources, including biomass. Disposal of waste PLA, such as other biopolymers, via anaerobic digestion (AD) to methane (as an energy carrier) is a forward-looking process, although it is currently problematic to implement. The research carried out to date proves that the decomposition of PLA in the AD process is difficult and essentially impossible under mesophilic conditions [10]. Some polymers are biodegradable under industrial composting conditions but not under anaerobic conditions. However, it should be emphasised here that the composting of biopolymers on biologically active landfills threatens emissions to the atmosphere [11]. A more correct direction, as evidenced by recent literature reports [12], is to study their disposal under anaerobic conditions. To date, researchers attempting to develop an effective technology for PLA decomposition in the AD process have used significantly elevated temperatures, i.e., hyperthermophilic and thermophilic reactor conditions, alkaline pre-treatment and co-digestion [13,14,15]. These measures have yielded good results; however, it must be borne in mind that the vast majority of biogas plants operate under mesophilic conditions and pre-treatment and the application of supporting commercial compounds generate additional costs, making implementation no longer economically viable. There is still a lack of information on the microbial population involved in the anaerobic digestion of biopolymers, including semicrystalline PLA [16,17].

In view of the aforementioned issues, Pilarska et al. (2022) suggested in their study the inclusion of PLA in mesophilic digestion as a cell carrier that is relatively durable under these conditions and improves the efficiency of the AD process [17]. The biopolymer residue (partially decomposed to lactic acid) in the digestate, after its application to the soil (as fertilizer), can act as a barrier to pathogenic bacteria and a carbon source for microorganisms [18,19]. From the point of view of sustainable agricultural development, environmental protection and research problems, this approach is optimal.

The changes taking place in the microbiome of anaerobically degraded matter are determined by the constant and changing environmental conditions during the process. They mainly include the lack of access to oxygen, reaction of the environment, temperature that depletes the medium, and additives, including cell carriers. Carrier materials strongly induce the dynamics of the dominant microbial population in every system [20,21]. Immobilised, selected, highly metabolic and surviving bacterial cells form a much more stable and synergistic microbiome, efficiently producing a biofilm. In the AD process, the decomposition of higher acids to acetic acid, carbon dioxide and hydrogen occurs at the acetogenesis stage [22,23]. Thermodynamically, this is one of the most difficult stages. This is because it requires the syntrophy of acetogens and methanogens, i.e., the so-called cross-feeding. In an anaerobic environment, syntrophy is an essential mutualistic cooperation (the so-called cross-feeding) between fatty acid-oxidising bacteria and methanogens, playing an important role in organic decomposition and methanogenesis [24,25]. It is acetogenesis that determines the amount of biogas produced. Acetate bacteria such as *Syntrophomonas* sp. and *Syntrophobacter* sp. play an important role here. They are responsible for converting acid fermentation products into acetates and hydrogen, providing the basis for methanogenic bacteria activity [26]. Their activity and development can be improved by the presence of carrier material.

This paper aims to identify the effect of the addition of granular polylactide on the growth rate and morphology of biofilm and genetic diversity of microbial communities in an anaerobic batch reactor (under mesophilic conditions), using modern analytical methods including next generation sequencing (NGS), the BioFlux microfluidic flow system and fluorescence microscopy. This article also presents key properties of the carrier material, including morphological and thermal properties, as well as the dynamics of changes in anaerobic bacterial abundance in the material collected from the bioreactors. An attempt was made to explain why the changes in microbial diversity, which occurred under the influence of PLA, improved methane generation efficiency in a reactor fed with substrates of similar composition [17].

## 2. Results and Discussion

### 2.1. Physicochemical Properties of the Carrier

**Figure 1:**
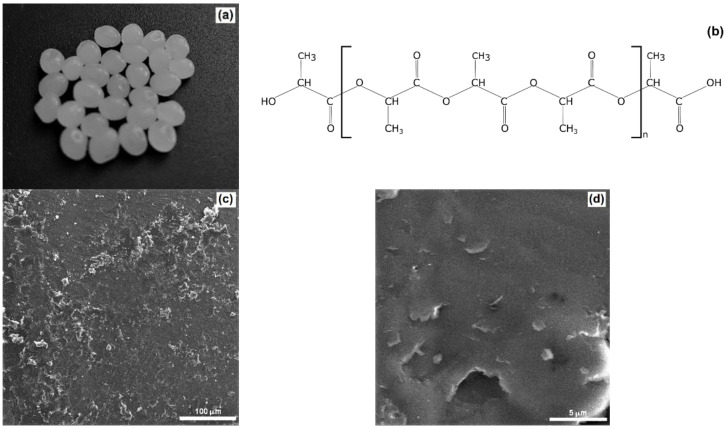
(**a**) Photograph of PLA granules, (**b**) structural formula of PLA and (**c**,**d**) SEM images of PLA at different magnifications.

In the first stage of the study, the physicochemical properties of PLA were analysed. The microstructural features of PLA granules (Figure 1a) were assessed using scanning electron microscopy, SEM (Figure 1c,d). The PLA sample had a relatively smooth and clear surface with furrows (Figure 1d) that provide some roughness to the granules. Similar SEM microscopic images can also be observed in other publications [27,28]. The elemental analysis results presented in the authors’ previous publication [17] confirm the presence—in PLA—of the highest mass fraction (56.34%) of carbon, slightly lower oxygen levels (40.84%) and the presence of silica at 2.83% (*w*/*w*).

**Figure 2:**
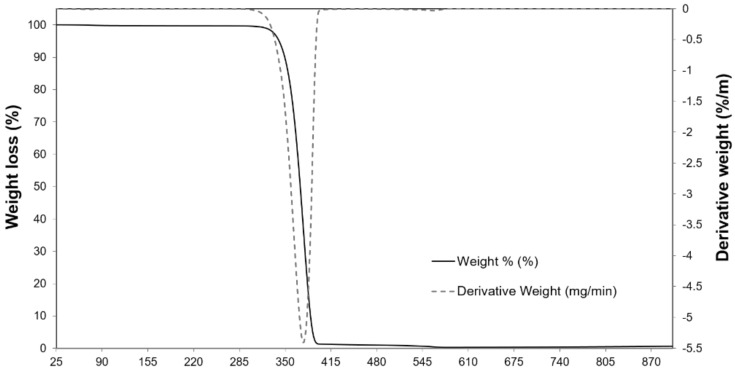
Thermogravimetric analysis (TGA)/differential thermogravimetric (DTG) analysis of PLA.

Figure 2 shows the results of the thermal analysis of PLA in the form of the thermogravimetric analysis (TGA) curves/differential thermogravimetric (DTG) curves. The figure illustrates the weight loss of PLA as a function of temperature. The results revealed that PLA is thermally stable in the temperature region below 285 °C, confirming its applicability in mesophilic anaerobic digestion as a cell carrier. This result is also confirmed by other literature data [5]. In turn, a temperature range of 150 °C to 200 °C is taken as the processing temperature for this material [29]. Polylactide (PLA) degrades completely in the presence of water. However, in the initial stage—hydrolytic degradation—PLA may not show moisture-induced weight loss due to the highly hydrophobic nature of this polymer.

### 2.2. Physicochemical Parameters and Total Bacterial Count of Digested Samples

The pH value is one of the most important parameters, being decisive to the course of organic matter decomposition because it affects both chemical reactions and activity of the bacterial flora [30]. The optimal pH for the growth of methanogens is 6.5–7.2 [17]. A decrease in the pH value in the system below 6.5 is caused by the accumulation of volatile fatty acids (VFA). Increased alkalinity affects the NH_3_ and NH^4+^ (ammonia anions) dissociation equilibrium. High pH and high temperature favour the accumulation of NH_3_(aq), which is a known process inhibitor [22]. The monitoring of the process stability should also include measurements of VFA and/or total alkalinity (TA) [30].

The values of monitoring parameters recorded in this experiment were within the ranges that did not inhibit the process. In the case of the CW–control sample, the following parameter ranges were obtained: pH, from 7.10 to 7.26; VFA/TA, from 0.38 to 0.25 and NH_4_^+^, from 966 mg·L^−1^ to 607 mg·L^−1^. For the CW + PLA sample, the ranges were as follows: pH, from 6.92 to 7.38; VFA/TA, from 0.43 to 0.26 and NH_4_^+^, from 1138 mg·L^−1^ to 1053 mg·L^−1^. The obtained values of these parameters not only confirmed the stable course of the methane fermentation process, but were also used to determine their correlation with the type of bacteria. These results are presented in Section 2.3.

**Figure 3:**
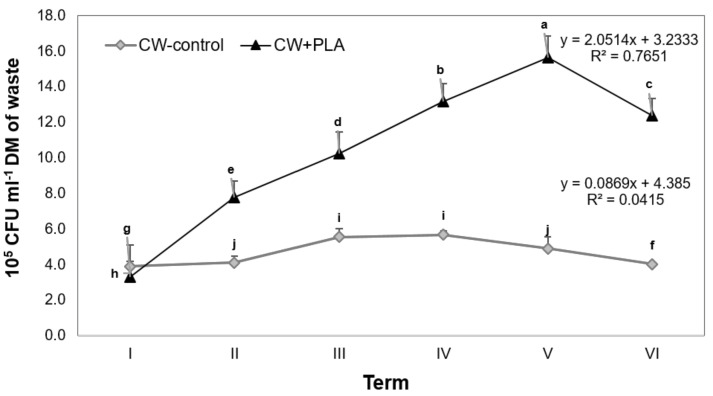
Total bacterial count changes found in digested sample material. Explanation: the same letter indicates a lack of significant differences (*p* < 0.05); CFU—colony-forming unit. Terms: I—2nd day; II—5th day; III—8th day; IV—12th day; V—16th day; VI—20th day of the process.

Due to its organic content, food waste is a substrate that allows an efficient anaerobic digestion process with the help of anaerobic bacteria [31]. Research by Pilarska et al. (2020) revealed that, in addition to the type and composition of the feedstock used for biogas production, the addition of the carrier used also significantly affected the abundance and biodiversity of the microbiome of digested waste [32]. Confirmation of these observations is provided by the results of our own study (Figure 3), which clearly show that the addition of a carrier in the form of PLA contributes to a statistically significant increase in the proliferation of anaerobic bacteria.

According to Liu et al. (2017), synthetic polymers are undoubtedly a very useful group of organic carriers due to their high chemical stability and mechanical resistance [33]. They have a high affinity for the cells of the immobilised microorganisms and enzymes, allowing them to bind persistently. Immobilisation of microbial cells on this type of carriers prevents their leaching and contributes to their density per unit volume of the reactor, resulting in better substrate utilisation and thus greater process efficiency [34].

The research revealed that, in addition to the type of experimental variant, the sampling date also significantly affects the level of anaerobic bacteria proliferation (Figure 3). This tendency appeared to be more pronounced in the digestate with PLA, which was confirmed via regression analysis. In the control variant, the abundance of the analysed microorganisms increased until the fourth (IV) time point, after which a decrease was recorded. In contrast, in the object of study with carrier addition, bacterial proliferation successively increased until the fifth (V) time point, after which it declined by 17.6%.

According to Choromański et al. (2016), determining the dynamics of changes in microbial abundance during methane fermentation is extremely important, as it allows the process to be controlled and provides information on when the methane production process breaks down [35]. Proliferation intensity of the methane fermentation microbiome is affected not only by the chemical composition of the substrate or the type of carrier introduced, but also by pH, temperature and inhibitory compounds present in the digested waste. The pH value as well as VFA/TA ratio are among the main environmental factors affecting the solubility of chemical compounds, their forms of occurrence, the degree of multiplication and the activity of the microbiome that is involved in methane fermentation [36,37].

### 2.3. Bacterial Community Abundance and Composition

In recent years, significant research has been carried out on the metagenomics of biogas-producing microbial communities, providing insights into their diversity and changes. Many new applications of bioinformatics have emerged in anaerobic digestion technology, offering great potential for optimising biogas/methane production, with the aim of achieving better process efficiency and stability [38].

**Figure 4:**
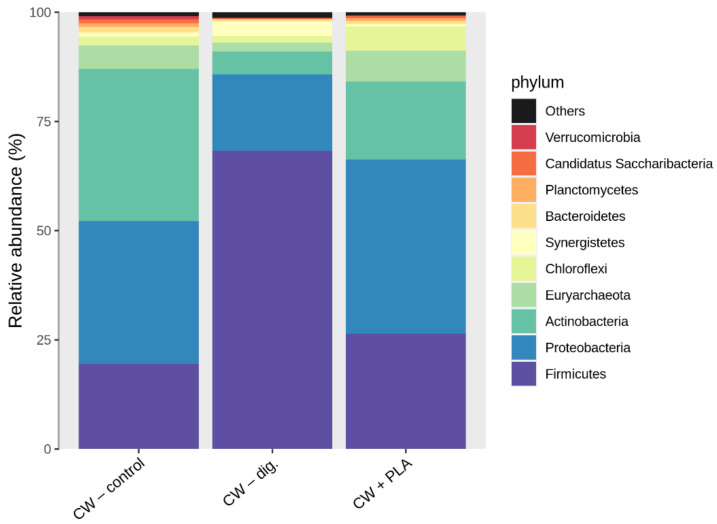
Composition of *phylum* taxa based on the metataxonomic analysis of the 16S rRNA gene.

The analysis of the bacterial sequencing data revealed the presence of 170 amplicon sequence variants (ASVs), 100 of which were identified at the genus level, belonging to 86 identified family taxa, 44 identified order taxa, 26 identified class taxa and 17 phylum taxa. The identified microorganisms belonged to bacteria (95% on average) and archaea (5% on average). *Clostridiaceae* and *Syntrophomonadaceae* were the most abundant as regards family taxa. Firmicutes were the most abundant phyla, followed by proteobacteria and actinobacteria (see Figure 4). The comparative analysis of the samples made it possible to examine the content of individual taxa over the course of the experiment. Actinobacteria were most abundant in the CW–control sample (34.87%), the percentage of which decreased to 5.3% in the CW–dig. sample and to 17.91% in the CW + PLA sample. At the same time, the dominant taxa in the CW–dig. and CW + PLA samples were firmicutes (CW–dig.: 68.27%, CW + PLA: 26.45%, CW–control: 19.45%). Interestingly, the number of proteobacteria decreased in the CW–dig. sample (17.47%) and increased in the CW + PLA sample (39.82%) compared to CW–control (32.70%).

Adequate proportions of actinobacteria and firmicutes were reported in digested systems with diatomaceous earth/peat (DEP) carriers [39]. In contrast, the change within the biodegradable sample with PLA addition, involving an increase in the proportion of proteobacteria compared to that in the CW–control, is indeed different compared to the system with DEP, where there was a decrease in this particular group of bacteria—both in the sample with the addition of the carrier and the sample without the addition of the carrier. Proteobacteria are the second largest group of CO hydroxyl oxidisers and consist of mesophilic and neutrophilic bacteria. Members of this group occur in different classes (alpha-, beta-, gamma- and epsilonproteobacteria) [40]. Proteobacteria were isolated from mesophilic environments, including freshwater, marine sediment, soil and anaerobic sludge; therefore, their elevated presence in a system with PLA may be indicative of favourable conditions for bacterial proliferation and activation. The improved environmental conditions may have contributed to an increase in the efficiency of the AD process as recorded during the biochemical methane potential (BMP) test [17].

**Figure 5:**
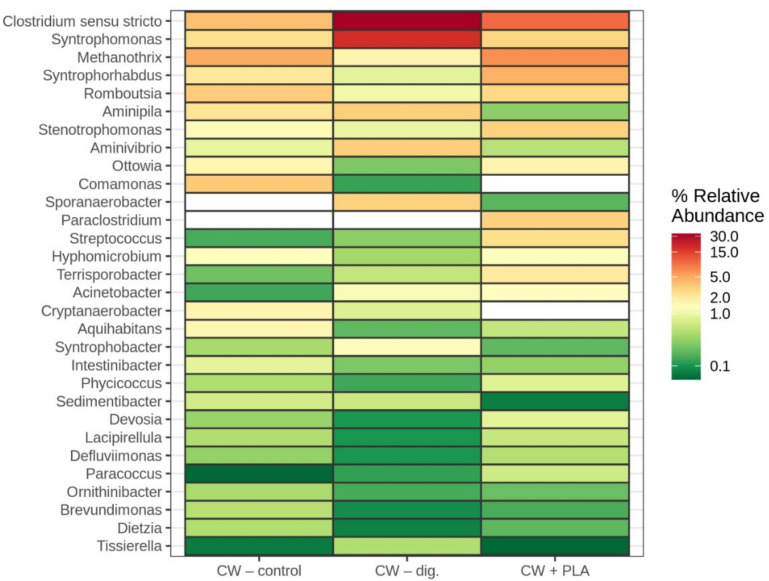
Composition of *genus* taxa based on the metataxonomic analysis of the 16S rRNA gene.

*Clostridium sensu stricto* was the most abundant genus identified (present in all samples), followed by *Syntrophomonas* (Figure 5). The number of *Clostridium sensu stricto* increased in the CW–dig. and CW + PLA samples by 2.5 and 8.5 times, respectively. In the case of *Syntrophomonas*, its content increased only in the CW–dig. sample (18.68%). The addition of PLA appears to have no effect on the growth of *Syntrophomonas*. This is different to the changes occurring in systems with DEP and with silica/lignin materials, in which bacteria of the genus *Syntrophomonas* predominated in the systems of confectionery waste digested with the aforementioned carriers [34,39]. There are many reports that have investigated the role of *Syntrophomonas* in anaerobic digestion and the importance of bacteria belonging to the *Syntrophomonadaceae* family during the methane production from LCFA (long chain fatty acid) and called this a syntrophic partnership with methanogens [39,41] The methane production from LCFA occurs through a syntrophic partnership between LCFA β-oxidising acetogenic bacteria and methanogenic archaea that are involved in maintaining low concentrations of acetate and hydrogen.

It should be mentioned that many unclassified sequences were also discovered in our study. The highest number of bacteria that were not identified to the genus level was in CW–control (58.061%). Its content decreased to 47.317% in the CW + PLA sample and to 23.856% in the CW–dig. sample. Most of those unclassified bacteria belonged to actinobacteria in CW–control and to proteobacteria in CW + PLA and CW–dig. samples. Compared to CW–control, there was a decrease in the number of 30 unclassified bacteria and an increase in the number of 8 unclassified bacteria after the experiment. There were no changes in the CW–dig. sample compared to CW–control for nine bacteria and in the CW + PLA sample for three unclassified bacteria.

For 15 unclassified bacteria, there was an increase in the CW + PLA sample and a decrease in the CW–dig. sample. The reverse was true for five bacteria, see Table 1. Table 1 shows data directly indicating that the addition of PLA affects changes in the microbiome relative to the process control and the digested sample without carrier addition.

The alpha (Table 2) and beta (Table 3) diversity indices calculated from 16S rRNA sequencing data showed no differences between the samples analysed. The sample with carrier addition (CW + PLA) was compared with the control sample (CW–control). Analysis of alpha and beta diversity showed no statistically significant differences. The values of Chao1, Shannon and Simpson indices for the compared samples were 127, 3.787, 0.955 for CW–control, and 125, 3.799, 0.965 for CW + PLA, respectively (see Table 2). The *p*-value for Bray–Curtis analysis (beta diversity) was 0.367 (see Table 3). These data indicate that there was no effect of carrier addition on biodiversity relative to the control sample.

**Figure 6:**
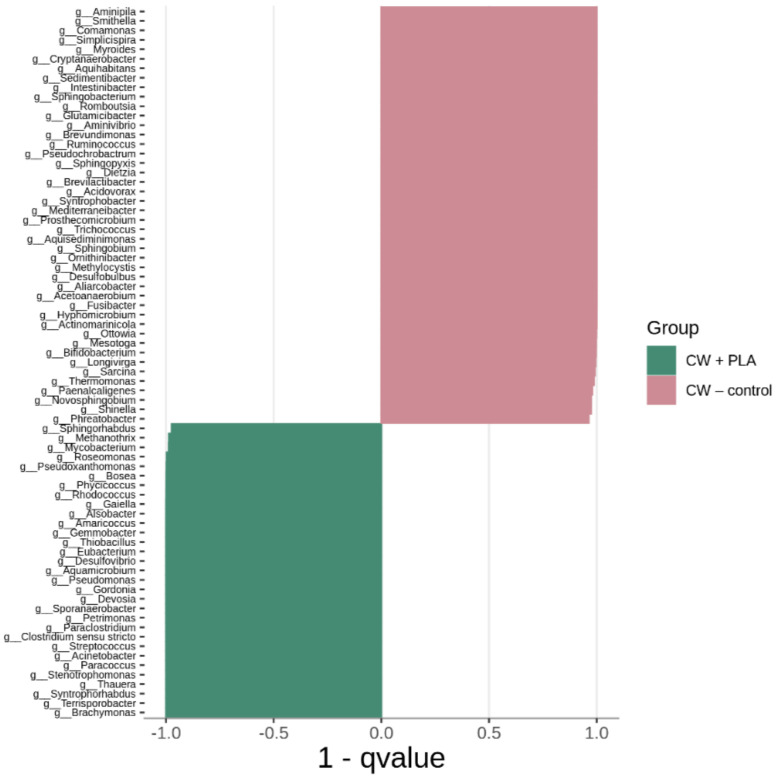
Comparison (MetaStat analysis) of bacterial genus composition between CW + PLA and CW–control.

**Figure 7:**
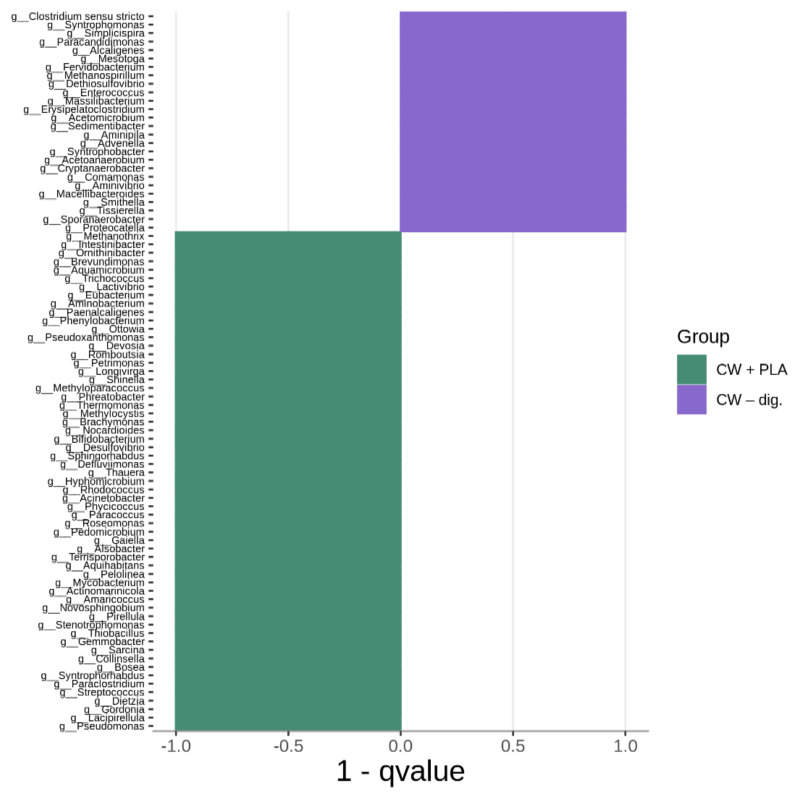
Comparison (MetaStat analysis) of bacterial genus composition between CW + PLA and CW–dig.

**Figure 8:**
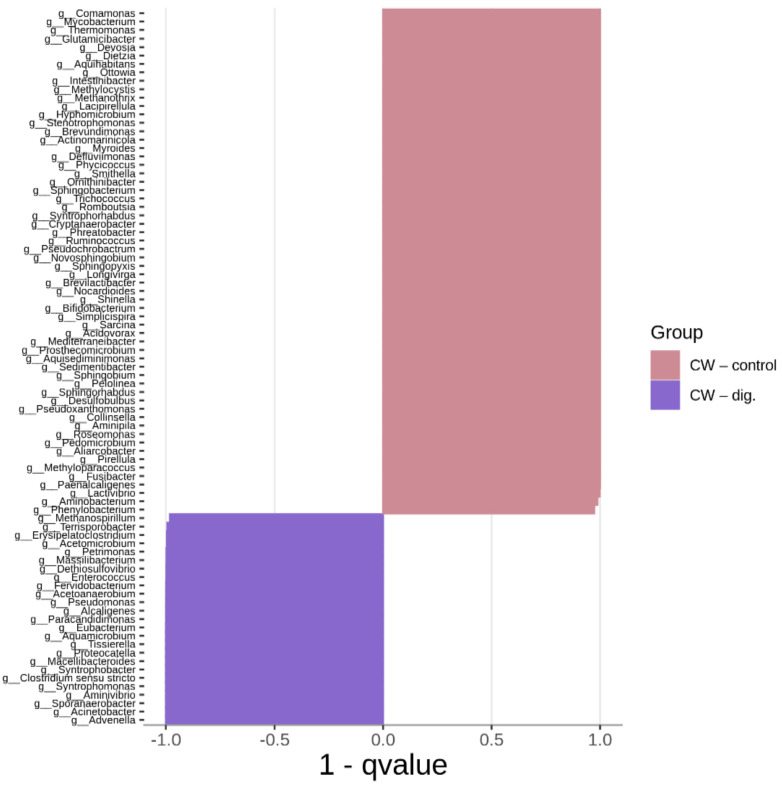
Comparison (MetaStat analysis) of bacterial genus composition between CW–control and CW–dig.

Figure 6, Figure 7 and Figure 8 show the differences in genus composition between all the samples analysed. The graphs indicate which taxa at the genus level occur in statistically greater amounts in the compared samples. Such analysis allows us to determine whether the addition of the carrier or the digestion process itself without the addition of the carrier causes changes in the microbiome relative to the control sample. This allows us to find bacteria that potentially affect the digestion process itself, and those whose growth is dependent on the presence of the carrier. We can also find bacteria that showed a decrease in abundance in the digested samples relative to the process control.

A comparative analysis between CW + PLA and CW–control samples showed differences in the abundance of 89 taxa (higher content of 35 bacteria in the CW + PLA sample).

The greatest increase in bacterial counts in CW + PLA compared to CW–control was observed for *Paraclostridium*, *Clostridium sensu stricto*, *Terrisporobacter*, *Acinetobacter*, and *Syntrophorhabdus*. A similar pattern was also observed by other researchers studying the effects of such additives to the anaerobic digestion system, using chitosan and activated carbon as support [42]. Almost the same number of statistically significant differences (85) was found when comparing CW + PLA with CW–dig. The genera *Ottowia*, *Romboutsia*, *Terrisporobacter*, *Stenotrophomonas*, *Syntrophorhabdus*, *Paraclostridium*, *Streptococcus* were the most prominent among 59 bacteria with a higher content in the sample with the addition of the carrier. The fermentation process without the addition of the carrier resulted in differences in the number of 88 genera of bacteria. However, the largest number of these differences was found for the CW–control sample (60). In one publication, *Syntrophorhabdus* was described as a mesophilic, absolutely anaerobic bacterium [43]. The type species is *Syntrophorhabdus aromaticivorans*, which is involved in the syntrophic oxidation of aromatic compounds such as benzoate into acetate. In syntrophic association with a hydrogenotrophic methanogen, this strain can use phenol, p-cresol, isophthalate, benzoate and 4-hydroxybenzoate.

**Figure 9:**
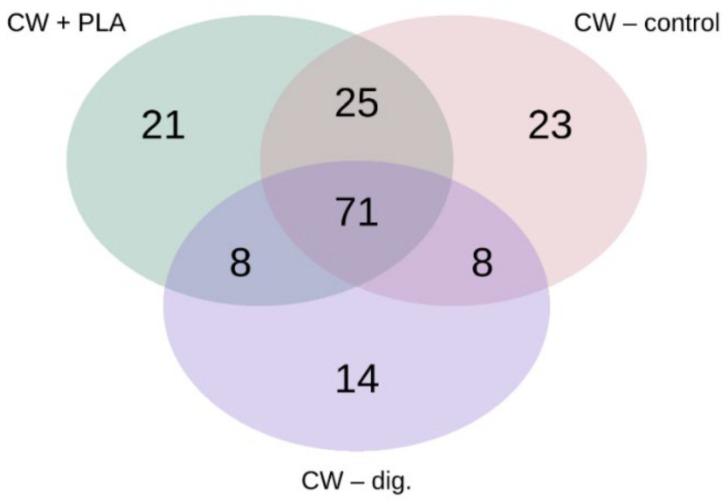
Venn diagram prepared based on the sequencing of the 16S rRNA gene. The data shown in the diagram refer to site-specific unique ASVs and the core microbiome.

The pooled analysis revealed an abundance of 58 unique bacterial ASVs, most of which were present in control samples, and the core microbiome was represented by 71 ASVs (Figure 9).

The unique bacteria present in CW + PLA belonged to the following genera: *Alsobacter*, *Amaricoccus*, *Bosea*, *Brachymonas*, *Desulfovibrio*, *Gaiella*, *Gemmobacter*, *Gordonia*, *Paraclostridium*, *Rhodococcus*, *Thauera*, *Thiobacillus*. The number of unique taxa represented 5.61% of all readings in CW + PLA, 2.7% in CW–control and 1.42% in CW–dig.

Therefore, it was the CW + PLA sample that exhibited the greatest quantitative changes in terms of an increase in bacterial counts compared to the CW–control (Figure 6) and CW–dig. (Figure 7) samples, as confirmed by the results of the overall analysis of the change in bacterial counts (see Figure 3). The addition of PLA enhances cell proliferation and activity up to the last period of the AD process, resulting in improved biodegradability of the matter and its higher methane production efficiency, as reported in the earlier study carried out by the authors [17]. However, it must be presumed that the addition of this polymer does not act as selectively as DEP [39]—the amount of unique taxa in the CW + PLA sample remained comparable to the CW–control (Figure 9). Furthermore, the results of the statistical analysis revealed no statistically significant differences (Table 2 and Table 3) between the three systems tested. Alpha diversity indices calculated from 16S rRNA sequencing data have very similar values (see Table 2), confirming no change in biodiversity levels. Bioinformatics analysis revealed the presence of a statistically significant positive correlation between pH and genus *Aminipila*, and a negative one with *Syntrophorhabdus*, *Devosia*, *Pseudoxanthomonas*, and *Roseomonas*. The VFA/TA ratio was negatively correlated with *Sedimentibacter*. Moreover, its value was positively correlated with the amount of 15 different genus of bacteria belonging to *Clostridia*, *Bacilli*, *Alphaproteobacteria*, *Betaproteobacteria*, *Bacteroidia*, *Actinobacteria*, *Deltaproteobacteria*, and *Thermoleophilia*. The level of ammonia anions, NH^4+^, was negatively correlated with *Aquihabitans*, *Ornithinibacter*, *Brevundimonas*, and *Trichococcus*. The significant impact of VFA/TA on the quality of the bacterial microbiome results from the specificity of anaerobic biodegradation, associated with changes in the proportion and availability of organic chemical compounds (as a bacterial medium).

Therefore, it can be concluded that the mechanism of the carriers’ effect on the cells is different depending on their chemical composition, microstructure or physical properties (such as conductivity). It is not excluded as a possibility that the PLA biopolymer may have acted largely as a stable cell carrier [17] but also as a high-value medium, creating conditions favourable for the growth of various bacterial genera.

### 2.4. Biofilm Formation Dynamics

The analysis of the dynamics of biofilm formation using the BioFlux microfluidic system shows a significantly faster increase in biofilm surface area for the CW + PLA sample from the 10th hour of observation onwards compared to the control (CW-control). These results correlate with those previously discussed, which consider an increased proliferation of bacterial cells in the variant with PLA addition.

**Figure 10:**
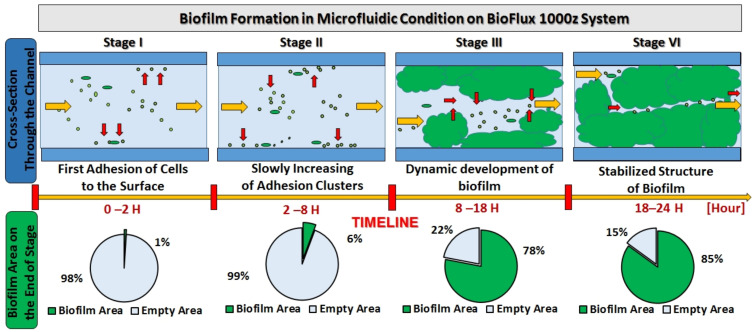
Stages of biofilm formation over 24 h in the channel of a microfluidic system (BioFlux 1000z).

Figure 10 shows the consecutive biofilm formation in a continuous flow in the environment of the CW + PLA sample. In four consecutive process steps, over a 24 h period, changes in biofilm structure are observable—from loose to dense and stable. According to a study carried out by Liu et al. (2022) on the effect of microfluidic channel geometry on biofilm formation, the rate of biofilm adhesion in the initial stage is determined by shear stress [44].

**Figure 11:**
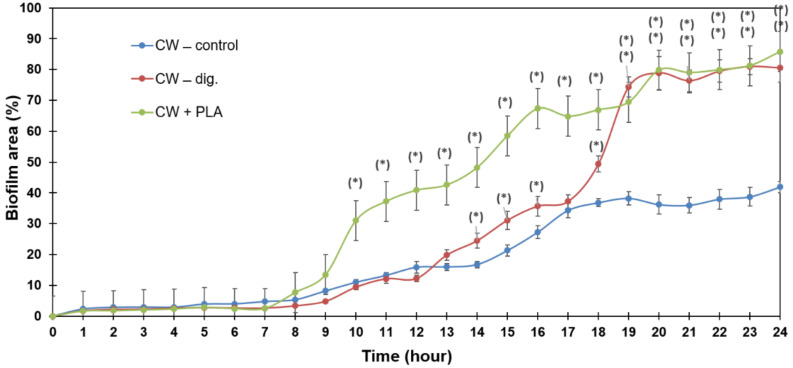
Dynamics of biofilm formation in the channel of the microfluidic system (BioFlux 1000z) for microbial samples from the bioreactor during 24 h of incubation under flow conditions (0.5 dynes/cm^2^); mean ± SD, *n* = 3; * statistically different from control *p* < 0.05.

Figure 11 shows the dynamics of biofilm formation for the three samples tested: CW–control, CW–dig. and CW + PLA. It should be noted that in the case of a digested sample but without the addition of the carrier (CW–dig.), there is not such a significant increase in the surface area of the biofilm formed in the channel (between 9 and 18 h). A similar level is only observed after 19 h compared to the sample with the addition of the carrier (CW + PLA). This implies that the carrier significantly promotes the initial adhesion of the microbiome and significantly accelerates its growth, which is positive. At the same time, it should be noted that both samples (CW–dig. and CW + PLA) achieve a significantly larger surface area of the biofilm produced (80%) compared to the control (CW), in which it is approximately 40%.

### 2.5. Visualisation of Microbiome

**Figure 12:**
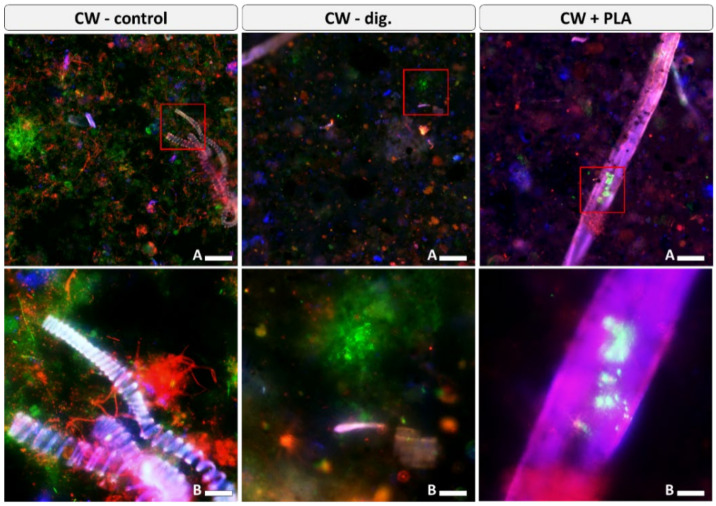
Visualisation of the microbiome of raw samples from the bioreactor. Blue/pink fluorescence—carrier with microorganisms; green fluorescence—live cells; red fluorescence—dead cells. Scale bar A = 100 μm (**up**) or B = 20 μm (**down**).

Visualisation of the microbiome of raw samples shows a highly variable structure of morphological forms of microorganisms (Figure 12).

For the control sample (CW–control), the highest biodiversity and potential for microbial accumulation was observed within the lignin-based microsprings. Imaging of the digested sample without the addition of the carrier (CW–dig.) shows a lower biodiversity compared to the control sample with a predominance of coccoid microorganisms. Images of the sample (CW + PLA) revealed a similar biodiversity to that of the CW–dig. sample. It was noted that quite numerous microbial consortia could form on sections of the carriers. This may imply that it is not so much the high biodiversity but the intensive growth of microorganisms and the formation of stable consortia on the surface of carriers that can significantly increase their efficiency.

The efficiency of the AD process is highly dependent on the dynamics of the microbial community, which is affected by biochemical, environmental and operational conditions. Metagenomic analyses, as applied to AD microbiome studies, provide insights into microbial community composition, dynamics of microbial growth, activity or functionality of microorganisms in response to operational changes. Research is mainly carried out with the goal of optmising AD for enhancing biomethanisation but also in order to achieve specific engineering goals, such as the co-mineralisation of recalcitrant pollutants and production of high value-added products through AD [45]. To date, it has been found, among other things, that MAG-related (metagenome-assembled genome) *Pelotomaculum* sp. and *Syntrophomonadaceae* sp. show a strong correlation with biogas production [46]. Their metabolic capacity confirmed the possibility of syntrophic interaction with methanogens via propionate oxidation [47]. Additionally, in this experiment, *Syntrophomonadaceae* were one of the most abundant bacteria based on family taxa. In the test presented in the authors’ earlier publication, it was the genus *Syntrophomonas* that was dominant in the sample with the addition of DEP, which was attributed to an improvement in the biodegradation of matter, as understood by the oxidation of LCFA and the decomposition of lower organic acids [39], and a consequent increase in methane production efficiency.

As evidenced by the results obtained for the biogas yield of samples digested with PLA [17], the application of this biopolymer as a cell carrier resulted in an increase of approximately 26% in the amount of methane produced under mesophilic conditions. This is a very favourable result, indicating an improvement to microbial function and changes in the microbiome. In the case of the DEP application, those were targeted changes, as much qualitative (modification of the microbiome) as they were quantitative (decrease in the number of taxa). When considering the effect of PLA on changes in the microbiome, there is mainly an increase in the number of bacteria in CW + PLA compared to CW–control and CW–dig. (Figure 6). More intense microbial proliferation may be indicative of the dual function of the organic polymer—support and medium. It is also interesting to note an increase in the number of proteobacteria, which are the most important phylum of Gram-negative bacteria, in relation to both the control and the digested system without the addition of the carrier (CW–dig.). However, in the case of the PLA application for AD process, there are no obvious changes in the level of biodiversity.

## 3. Materials and Methods

### 3.1. Materials

In this study, the feedstock for biogas production was mixed confectionery waste (CW), sourced from a production facility near Poznań, Wielkopolskie Voivodeship, Poland. Digested sewage sludge (SS), taken from the municipal sewage treatment plant in Poznań, was used as inoculum. This SS can act as a buffer in the digested system, which is particularly important during carbohydrate decomposition [48]. In the experiment, polylactide (PLA) was used as a cell carrier. It is a granular biopolymer produced by Nature Works LLC, Plymouth, MA, England, with the grade name Ingeo 2500HP [17].

Table 4 shows selected values for the physicochemical parameters of the substrate and inoculum.

### 3.2. Bioreactor Configuration and Operation

The first stage of the experiment consisted of preparing fermentation mixtures in the form of two batches: CW–control, CW + PLA (Table 5). The digestion mixture ratios were based on the Verein Deutscher Ingenieure (VDI) 4630 guidelines [49], which concern the digestion of organic materials, substrate characterisation, sampling, material data collection and fermentation tests. According to these guidelines, the batches were prepared so that their total solids (TS) content was kept below 12% to ensure adequate mass transfers. The low content of total solids in SS (Table 4) ensures that the pumpability of the suspended matter in the technical installation and the correct rheological parameters are maintained [50]. The volatile solids (VS) content ranged from 1.5 to 2% in the batches, while their pH was neutral. Table 5 shows the compositions and selected parameters of the mixture.

Anaerobic digestion (AD) was carried out in a multi-chamber batch bioreactor, the scheme of which was discussed in the author’s earlier publications [30,51]. The process was carried out under mesophilic conditions (38 °C). The total number of digestion tanks in the study was six (each sample was tested in triplicate). The batch in bioreactors with a capacity of 1.0 L was subjected to agitation once a day. The hydraulic retention time (HRT) of the process was 21 days. According to German DIN 38 414-S8 (DIN standard, Deutsches Institut für Normung) [52], the experiment was carried out until the daily biogas production fell below 1% of the total biogas produced, in all bioreactors. The volume of biogas produced was measured every 24 h. The concentration of methane, carbon dioxide, hydrogen sulphide, ammonia and oxygen in the biogas was measured using a Geotech GA5000 gas analyser (Geotech, Coventry, UK). The gas concentration was measured with Mg-72 and Mg-73 measuring instruments by Alter inc., Tarnowo Podgórne, Poland. Estimates of biogas yields (in m^3^ Mg^−1^), in terms of total solids and volatile solids, were made based on experimental data. The cumulative amount of biogas—including methane—obtained from the individual samples was calculated based on the formulas presented in the authors’ earlier studies [30,51].

### 3.3. Physicochemical Analysis

The feedstock, inoculum, prepared batches and digested samples were subjected to pH (potentiometric analysis) and electrolytic conductivity measurements using Elmetron CP-215 (ELMETRON, Zabrze, Poland). Their total solids (TS) were determined by drying at 105 °C (Zalmed SML dryer, Zalmed, Łomianki, Poland) and volatile solids (VS) via combustion at 550 °C (MS Spectrum PAF 110/6 furnace, MS Spectrum, Warsaw, Poland)—gravimetric analysis. The carbon content of these materials was via by combustion at 900 °C followed by CO_2_ determination (Infrared Spectrometry, OI Analytical Aurora 1030W TOC Analyzer, Picarro Inc., Santa Clara, CA, USA). Moreover, the nitrogen content analysis—titration was performed—, as well as the Kjeldahl method using 0.1 N HCl, in the presence of Tashiro’s indicator and ammonium nitrogen—the distillation and titration method using 0.1 N HCl, in the presence of Tashiro’s indicator [39]—were also employed. To determine the concentration of VFA (volatile fatty acids), TA (total alkalinity), and the VFA/TA ratio (volatile fatty-acids-to-total-alkalinity ratio) in the digested samples, 5 mL of a given sample was collected, then titrated using 0.1 N of sulphuric acid solution (H_2_SO_4_) up to pH 5.0 to calculate the TA value. The VFA value was obtained after a second titration step between pH 5.0 and pH 4.4.

The PLA morphology and microstructure were studied on SEM (scanning electron microscope) images captured with an FEI Quanta FEG 250 microscope (Thermo Fisher Scientific, Waltham, MA, USA). The microscope was operated in low-vacuum mode at a pressure of 70 Pa and an accelerating voltage of 10 kV. Prior to testing, samples were coated with gold for 5 s using a Balzers PV205P coater (Balzers, Switzerland) [53].

The PLA thermal stability was analysed using a TGA4000 Thermal Analyser (Perkin Elmer, Waltham, MA, USA). The tests were carried out in a nitrogen atmosphere. Samples were heated from 25 °C to 900 °C in a nitrogen flow (20 mL·min^−1^). They were held at 995 °C for 1 min and then cooled.

### 3.4. Microbial Analysis

#### 3.4.1. Analysis of the Total Bacterial Count

Analyses performed using selective standard agar by Merck (Darmstadt, Germany) allowed the measurement of colony-forming units (CFU) of anaerobic bacteria (AnB). Bacterial population counts were recorded after 24 h of incubation at 35 °C [39]. The anaerobic conditions in which Petri dishes were incubated were maintained using the Anaerocult anaerobic incubation system (Merck, Darmstadt, Germany).

#### 3.4.2. DNA Extraction and Next Generation Sequencing (NGS)

Total DNA was extracted from 500 mg of each sample using the Genomic Mini AX Soil kit (A&A Biotechnology, Gdynia, Poland) according to the manufacturer’s instructions. The extracted DNA was quantified using the Quant-iT HS ds.-DNA assay kit (Invitrogen, Carlsbad, CA, USA) on a Qubit2 fluorometer (Invitrogen); 2 µL of extracts were analysed using a 0.8% agarose gel. The metagenomic analysis was based on the V3-V4 hypervariable region of the 16S rRNA gene. Specific primers 341F and 785R were used for amplification of this region and library preparation. The polymerase chain reaction (PCR) was performed using the Q5 Hot Start High-Fidelity DNA Polymerase Kit (NEB Inc., Ipswich, MA, USA) under reaction conditions according to the manufacturer’s specifications. The sequencing was performed by means of a MiSeq sequencer in 2 × 250 bp paired-end (PE) technology, 144,429.7 of 23 using Illumina v2 chemistry kit [34,39]. The reactions were performed according to the Illumina V3-V4 16S RNA amplification protocol (Illumina, Inc., San Diego, CA, USA), while sequencing was carried out according to Illumina MiSeq PE300 software (Genomed S.A., Warsaw, Poland). The automated data analysis was performed on MiSeq and in Illumina’s Cloud BaseSpace environment, using the 16S Metagenomics protocol (ver. 1.0.1). The libraries were prepared in a similar manner according to the attached Illumina protocol.

### 3.5. Bioinformatics and Statistical Analysis

The DADA2 (1.14) package [54] in R software (3.6.0) [55] was used for processing demultiplexed fastq files. Based on the quality graphs, the last 20 forward and 20 reverse bases were truncated (filter parameters: maxN = 0, maxEE for both reads = 2, truncQ = 2). The subsequent analysis steps included learnErrors (error rate estimation), dada (exact sequence variants), removeBimeraDenovo (removal of chimeric sequences). The latest version of the modified Ribosomal Database Project (RDP) v18 [56] was used for taxonomy assignment using IDTAXA [57]. The results were implemented into the phyloseq (1.22.3) package [58] (chloroplast or mitochondrial DNA was deleted).

### 3.6. Microfluidic Flow System BioFlux 1000z

The samples were prepared as follows: for each sample (CW–control: sample at the first step of the process, not digested and at the last AD stage; CW–dig., digested; and CW + PLA, digested with PLA), 2 mL of sludge was collected from the bioreactor. The supernatant was then centrifuged (5000 RPM, 5 min), retained and used as brain heart infusion broth (BHIB, Biomaxima, Lublin, Poland) culture medium.

Firstly, the samples were heavily vortexed to enrich liquid fraction with the highest possible amount of bound microorganisms. Then, the starting density for all test samples was established by adding the liquid fraction of the same optical density to BHIB culture medium. Thus, the potential of the microbial population for the tested samples to form a biofilm over time under flow conditions was determined.

Standardised test suspensions of 0.8 OD were prepared by collecting 100 μL of raw sample and suspending it in the appropriate volume of BHIB. Initially, the inoculum (1 mL) was incubated without flow to achieve sample adhesion, the wells were filled and a flow rate of 0.5 dynes per 1 cm^2^ was initiated. The plate was incubated at 28 °C for 24 h in an incubator chamber (Carl Zeiss Pecon Incubator XL S1, Mannheim, Germany). In the environmental chamber, the gas compositions recommended for the anaerobic chamber (nitrogen 80% and carbon dioxide 20%) were used for achieving the closest to optimal anaerobic conditions during incubation. Parameters such as flow shear, adhesion time and inoculum concentration were tested for biofilm production in the microfluidic system. Imaging was performed at 1 h intervals (every 1 h for 24 h; three images per channel at different positions) using an Axio Inverted Observer 7 fluorescence microscope (Carl Zeiss, Germany) equipped with an Orca Flash 40 camera (Hamamatsu, Japan) and 20× lens (differential interference contrast [DIC] mode). The images were then analysed using Image J software based on background threshold analysis [59]. The tests were conducted in three biological replicates and three technical replicates.

### 3.7. Microscopic Visualisation of Microbiome

Direct microscopic preparations of bioreactor samples were made. For the CW–control, CW–dig. and CW + PLA samples, they were prepared by adding 100 μL of each sample from the bioreactor to 900 μL of PBS with fluorescent dyes (1 μL Syto 9 [Ex λ = 488 nm] and 1 μL of propidium iodide [Ex λ = 543 nm] [PI]). A filter for DAPI (4′,6-diamidino-2-phenylindole) (filter Ex λ = 359 nm) was used for visualisation of the carriers. The samples were incubated for 30 min under anaerobic conditions and then rapidly visualised by making microscope slides using two typical pieces of glass [60]. The samples were analysed using a fluorescence microscope (magnification: 10× and 40×) with an Olympus BX51 + DP25 camera (Olympus, Shinjuku, Tokyo, Japan). Scale bar A = 100 μm and B = 20 μm.

## 4. Conclusions

The premises, which have been formulated based on the thermal analysis results, indicate that granular PLA can successfully act as a stable cell carrier under mesophilic conditions. The results of the microbiological analysis of the total bacterial count, which was carried out during the AD process, revealed a successive multiplication of bacteria up to the last sampling date in the object of study with PLA addition. The analysis of changes in genetic diversity of bacterial communities, which was performed using next generation sequencing (NGS), proved both the quantitative and qualitative changes in the bacterial microbiome that occur as a result of anaerobic biodegradation.

Actinobacteria was the most abundant type in the CW–control sample (34.87%), while firmicutes dominated in digested samples (CW–dig. and CW + PLA). Interestingly, the number of proteobacteria decreased in the CW–dig. sample (17.47%) but increased in the CW + PLA sample (39.82%) compared to the CW–control sample (32.70%). This phenomenon demonstrates the existence of favourable conditions in the AD environment for the proliferation and activation of bacteria. On the other hand, the genus-level analysis shows another phenomenon that is unusual for the carriers that were previously tested by the authors—the lack of effect of the PLA addition on the growth of *Syntrophomonas*. Polylactide (PLA) clearly has an effect on the intensification of bacterial proliferation; however, it does not fundamentally change microbiome biodiversity, which was also confirmed via statistical analysis. The different direction of PLA interaction is most likely related to physicochemical properties of the biopolymer, including its chemical composition and microstructural properties. Hypothetically, PLA under mesophilic process conditions primarily acts as a carrier but also as a high-value medium that enhances microbial activity and growth. This effect contributes directly to an increase in the efficiency of biogas production. An improvement to the dynamics of biofilm formation, under the effect of PLA, was reported in the test using the BioFlux microfluidic system. The formation of bioaggregates in the CW + PLA sample was observed using fluorescence microscopy—the images taken showed carrier sections that were clearly covered with microbial consortia. PLA degradation was measured over time in the anaerobic reactor.

The above statements prove that waste PLA can successfully function as a microbial carrier in mesophilic conditions, which are the most common in biogas production (on a technical scale). This proposal is an alternative method for the sustainable use of this biopolymer. In the near future, the authors plan to also carry out a study on the degradation of PLA and its composites over time in the anaerobic reactor.

## Figures and Tables

**Table 1:** Changes in the abundance of unclassified bacteria in CW + PLA and CW–dig. samples compared to CW–control (↓—decrease after the experiment; ↑—increase after the experiment; ≈—same amount after the experiment; – indicates no number of analysed bacteria in control or sample after the experiment).

	CW + PLA	CW–dig.		CW + PLA	CW–dig.
Unidentified_003	↑ 0.4	↓ 2.7	Unidentified_040	↑ –	≈ –
Unidentified_006	↑ 0.2	↓ 3.6	Unidentified_042	↑ 0.4	↓ –
Unidentified_007	↑ 0.7	↓ 1.1	Unidentified_043	↑ –	≈ –
Unidentified_010	↑ 0.9	↓ 5.8	Unidentified_046	↑ –	≈ –
Unidentified_011	↑ 0.6	↓ 2.5	Unidentified_047	↑ –	≈ –
Unidentified_014	↓ 5.9	↑ 0.2	Unidentified_048	↓ 2.1	↑ 0.6
Unidentified_016	↑ 0.9	↓ 3.4	Unidentified_049	↑ –	≈ –
Unidentified_019	↑ 0.9	↓ 3.4	Unidentified_050	↑ –	≈ –
Unidentified_022	↓ 2.3	↑ 0.5	Unidentified_056	↑ –	≈ –
Unidentified_024	↑ 0.6	↓ –	Unidentified_057	↑ –	≈ –
Unidentified_025	↓ 1.1	↑ 0.5	Unidentified_061	↑ 0.6	↓ –
Unidentified_028	↑ 0.7	↓ 4.0	Unidentified_064	↑ –	≈ –
Unidentified_029	↑ 0.6	↓ –	Unidentified_069	↑ 0.6	↓ –
Unidentified_038	↑ 0.5	↓ –	Unidentified_070	↓ 2.2	↑ 0.4
Unidentified_039	↑ 0.9	↓ –			

**Table 2:** Alpha diversity indices calculated from 16S rRNA sequencing data.

	Chao1	Shannon	Simpson
CW–control	127	3.787	0.955
CW–dig.	101	2.857	0.846
CW + PLA	125	3.799	0.965

**Table 3:** Beta diversity index (Bray–Curtis analysis) calculated from 16S rRNA sequencing data.

	CW–Control	CW–dig.
CW–dig.	0.649	–
CW + PLA	0.367	0.606

**Table 4:** Substrate and inoculum characteristics.

Materials	pH	Cond.	TS	VS	C/N Ratio	C	N	N-NH_4_
	−	(mS cm^−1^)	(wt %)	(wt %_TS_)	−	(wt %_TS_)	(wt %_TS_)	(wt %_TS_)
CW	7.01	3.29	94.85	98.31	49.90	45.91	0.92	0.28
Inoculum	6.94	28.62	2.96	78.65	3.20	26.72	8.34	3.96

CW—confectionery waste; Cond.—conductivity; TS—total solids; VS—volatile solids.

**Table 5:** Composition and selected properties of substrate/inoculum batches.

Batches	WF (g)	Carrier (g)	Inoculum (g)	pH	TS (%)	VS (%)
CW–control	10.6	−	900.0	6.95	4.23	70.15
CW + PLA	10.6	20.0	900.0	7.08	4.05	67.31

## Data Availability

Not applicable.
